# Control Strategies for Scabies

**DOI:** 10.3390/tropicalmed3030098

**Published:** 2018-09-05

**Authors:** Daniel Engelman, Andrew C. Steer

**Affiliations:** 1 Tropical Diseases, Murdoch Children’s Research Institute, Parkville VIC 3052, Australia; Andrew.Steer@rch.org.au; 2 Department of Paediatrics, University of Melbourne, Parkville VIC 3052, Australia; 3 Department of General Medicine, Royal Children’s Hospital, Parkville VIC 3052, Australia; 4 International Alliance for the Control of Scabies, Parkville VIC 3052, Australia

**Keywords:** scabies, neglected tropical diseases, impetigo, mass drug administration, ivermectin

## Abstract

Scabies is a neglected tropical disease of the skin, causing severe itching and stigmatizing skin lesions. Further, scabies leads to impetigo, severe bacterial infections, and post-infectious complications. Around 200 million people are affected, particularly among disadvantaged populations living in crowded conditions in tropical areas. After almost 50 years, research into scabies control has shown great promise, particularly in highly-endemic island settings, but these findings have not been widely adopted. Newer approaches, utilizing ivermectin-based mass drug administration, appear feasible and highly effective. Inclusion of scabies in the WHO portfolio of neglected tropical diseases in 2017 may facilitate renewed opportunities and momentum toward global control. However, further operational research is needed to develop evidence-based strategies for control in a range of settings, and monitor their impact. Several enabling factors are required for successful implementation, including availability of affordable drug supply. Integration with existing health programs may provide a cost-effective approach to control.

## 1. Introduction

**Figure 1:**
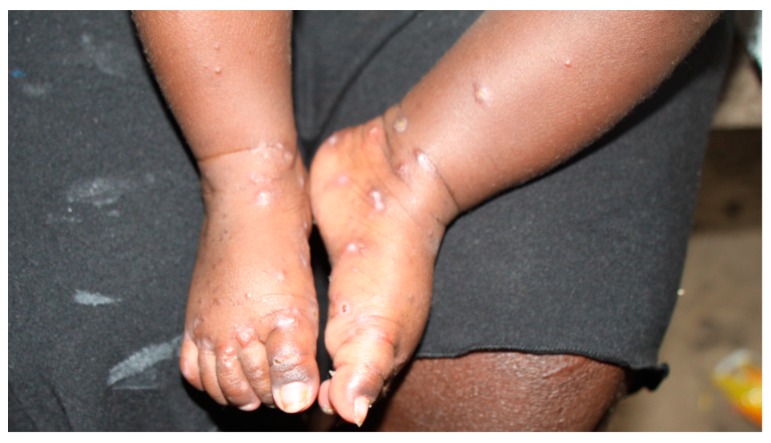
Infant with typical rash of scabies.

Scabies is a skin disease caused by infestation with the mite, *Sarcoptes scabiei* var. *hominis.* The female mite, measuring less than 0.5 mm, burrows into the skin, where antigens on the exoskeleton of the mite, along with its saliva, excreta, and eggs, elicit a hypersensitivity reaction [1]. The resulting skin lesions most commonly affect the hands, wrists, ankles, and feet (Figure 1). In the vast majority of cases of common scabies (also known variably as ordinary, classical, or typical scabies) there is a low number of mites on the patient’s body (5 to 15). Crusted scabies (formerly known as Norwegian scabies) is a rare form of the disease characterized by hyperinfestation with thousands to millions of mites and hyperkeratotic ‘crusted’ skin [2].

Transmission of scabies is predominantly via skin-to-skin contact. Transmission from bedding or clothes is rare in ordinary scabies, but can occur in crusted scabies because of its tremendous mite burden. The risk of transmission of scabies increases with higher levels of population density, reflected by the high endemicity observed in communities living in poverty with associated crowded housing conditions, and by outbreaks in in residential care facilities, prisons, schools, and refugee camps. Patients with underlying immunodeficiency from any cause, such as human immunodeficiency virus, human T-lymphotropic virus type 1 or corticosteroid treatment, or those with neurological conditions, are at an increased risk of crusted scabies [2,3,4].

Scabies causes considerable suffering due to the intense itch and associated scratching, leading to sleep disturbances which, in turn, impact on school and work attendance and performance, and ultimately the economic productivity of whole communities [5]. Like many infections that affect the skin, scabies is associated with social stigma and leads to social exclusion because of the appearance of the affected individual and attendant feelings of shame, as well as fears within the community of spread of infection [5,6]. Scabies can have a marked impact on quality of life measures, similar to the recognized psychological impact caused by other skin conditions such as psoriasis and vitiligo [7].

In endemic settings, scabies lesions are often super-infected with the bacteria *Streptococcus pyogenes* and *Staphylococcus aureus.* Bacterial infection occurs due to breaches in the skin barrier as a result of mite burrows and associated scratching [8], as well as direct effects of the scabies mite in downregulating host immunity and optimising conditions for bacterial growth [9,10]. These bacteria can cause local soft tissue infections such as impetigo, cellulitis, and abscesses, and can lead to life-threatening diseases including sepsis, and in the case of *S. pyogenes*, post-streptococcal glomerulonephritis [11], and possibly acute rheumatic fever (this link is epidemiologically associated but, as yet, unproven) [12,13].

This review aims to re-examine the rationale for public health control of scabies, evaluate the available evidence for scabies control interventions, and identify the barriers and future research priorities needed to develop and scale-up implementation of scabies control activities.

## 2. Burden of Disease

Scabies occurs worldwide, and is estimated to affect over 200 million people at any single time [14]. The highest prevalence of scabies occurs in tropical areas, especially in disadvantaged populations, and especially among children and the elderly. The Global Burden of Disease (GBD) study reported that scabies directly accounts for 0.21% of global disability-adjusted life years [15]. However, there are substantial gaps in our knowledge of the epidemiology of scabies and its complications.

First, many countries have no available data on scabies prevalence, including countries where disease burden is likely to be high, based upon known risk factors (poverty and overcrowding). For example, in a systematic review of prevalence studies of scabies published 1985 to 2015, only five sub-Saharan African countries were represented among the 48 studies included [16]. Where data do exist, they may be outdated and not representative of the current burden.

Second, routinely-collected data, and therefore GBD data, do not reflect actual disease burden. In countries where community-based prevalence survey data are available, the prevalence of scabies observed in these surveys is frequently substantially higher than the burden reported at the clinic level, and by the GBD study. For example, in the Solomon Islands, routine Ministry of Health clinic data identified 4759 cases of scabies in 2016 (total population of 584,000, corresponding to an incidence of 0.79% per year), in contrast to a detailed community-based survey in one province in the same year in which the prevalence was 19.2% [17]. This discrepancy may be because individuals within communities may not present with scabies (perhaps because it is ‘normalized’ or because effective treatments are often unavailable), or because there is under-diagnosis at clinics when patients do present [18].

Third, even when prevalence surveys are conducted, there is substantial heterogeneity in design and methods across studies. A key issue is the method used to diagnose scabies, which ranges from recovery of live mites by scraping of the skin, to a clinical diagnosis based on varying combinations of diagnostic features. This variability, noted even among scabies treatment trials [19], limits comparison of disease burden estimates across studies.

Fourth, there is limited evidence to determine the magnitude of the association between scabies and its complications. Available epidemiologic data suggest that, in highly endemic settings, at least 40% of impetigo lesions can be attributed to scabies, and an even higher proportion among younger children (71% in one study) [17,20,21,22]. The high attributable risk observed in epidemiologic studies is supported by trials of community control of scabies, as outlined below, where impetigo prevalence has been shown to fall consistently and substantially with reductions in scabies prevalence.

## 3. The Need for Surveillance and Control

The World Health Organization Department of Neglected Tropical Disease (NTD) Control designated scabies as an NTD for large-scale disease control action in 2017. This designation was in recognition of: (1) the large known burden and impact of scabies, justifying a global response; (2) the geographic distribution of scabies among disadvantaged populations living in tropical and subtropical regions; (3) the amenability of scabies to public health control efforts, especially noting the success of mass drug administration (MDA) for scabies (detailed below); and (4) the broad neglect of scabies across multiple research domains [23]. In 2018, the ninth meeting of the WHO NTD Strategic Technical Advisory Group Global Working Group on Monitoring and Evaluation of NTDs recognized that there is a priority need to develop resources to aid defining the global burden of scabies, and to provide guidance for countries and regions for public health approaches for scabies control, including in outbreak situations [24].

Guidance is required for assessment of disease burden at local, national and regional levels, and should include guidelines for standardised diagnosis and surveillance methodology. Consensus criteria for the diagnosis of scabies were recently developed using a four-round Delphi process among 34 international experts, under the auspices of the International Alliance for the Control of Scabies (IACS) [25]. The IACS Criteria include three levels and eight subcategories such that the criteria can be applied across a range of situations, from a dermatologist’s office to a field survey in a resource-limited setting (Table 1) [26].

The approach to community control for scabies is best aligned with the core strategies utilized by WHO Department of NTD Control: preventive chemotherapy using MDA, and/or innovative and intensified case management. Guidance around which approach to use will depend upon a number of factors, particularly disease burden at the community level. Therefore, feasible and accurate assessment of scabies prevalence will be crucial to inform an appropriate public health response, and also to monitor the effectiveness of this response. Below we outline the evidence to inform community control strategies for scabies, particularly MDA.

## 4. Community Control 

The first reported public health initiatives to control scabies came from the San Blas islands (now known as the Guna Yala region) of Panama in the 1970s and 1980s. Scabies was introduced to the Guna populations and rapidly became endemic, with reported prevalences of 40–70% [27]. In a landmark series of studies, Taplin and colleagues compared approaches to scabies control in the islands. They found that treatment of all community members with 1% lindane resulted in a 98% cure rate at 10 weeks, whereas treatment of only those with clinically evident scabies had a lower effectiveness of 50% [28]. In areas where the entire community could not be treated, scabies was noted to return rapidly, leading the authors to conclude that, in those highly-endemic settings, ‘treatment of individual patients without regard to community epidemiology is time consuming, and unlikely to have a significant impact in epidemic situations’ [27]. Suspicion of developing mite resistance and the adverse effects profile of lindane then prompted the investigation of topical permethrin 5% for 756 residents on the island of Ticantiki. Following a MDA campaign with very high coverage, new arrivals to the island were treated and ongoing surveillance identified and treated new cases [27]. Scabies prevalence was reduced from 32% to below 2% and maintained for 3 years. Bacterial skin infection (impetigo) also declined from 32% to l% in children without use of antibiotics. The permethrin-based MDA was well tolerated with few adverse events, but required a large project team for implementation. The team used directly observed applications, including to genital and breast areas, which may not be acceptable by some communities.

Drawing on the work in Panama, a control program commenced in five small, densely-populated islands in the Solomon Islands from 1997 to 2000 [29]. Use of topical treatments in this setting was considered ‘so difficult as to be unacceptable and impractical’. Reports of the effectiveness of ivermectin for the individual and mass treatment of scabies [30], and knowledge of safety as a MDA strategy for lymphatic filariasis and onchocerciasis [31,32], led to selection of oral ivermectin as the main treatment, offered to the whole population as MDA. Permethrin 5% cream was given to children weighing less than 15 kg and pregnant women. Henceforth, we refer to this strategy of using topical treatments for ivermectin-contraindicated groups as ‘ivermectin-based MDA’. Children (but not adults) were then re-examined at 6-monthly intervals and those with scabies and their household contacts were re-treated. Returned travelers and visitors were also treated, even if asymptomatic. No additional antibiotics were used. Over the three years of observation, the prevalence of scabies in children reduced from 25% to less than 1%. The proportion of children with open sores, the median number of sores, and the proportion of children with microscopic hematuria also reduced significantly. In contrast to the Panama experience, where scabies resurgence was observed after disruption of permethrin supply, when 338 residents of the same communities in the Solomon Islands were examined for scabies more than 15 years later, only a single case of scabies was found [33]. 

An ecological study from northern Australia aimed to reduce scabies and secondary bacterial skin infection in five remote communities from 2004 to 2007 [34]. Permethrin 5% was offered annually to all community members, but application was not directly observed. Children aged less than 15 years were screened regularly and referred for treatment if scabies or impetigo were identified. Whereas the prevalence of bacterial skin infection reduced during the project, there was little effect on scabies. A nested study found that actual use of permethrin was low, with only 44% of contacts applying the cream. Those households that did apply the cream had a much lower rate of scabies transmission [35].

The first controlled study of MDA for scabies control was conducted in Fiji in 2012. Three small island groups were randomized to one of ivermectin-based MDA (with permethrin for contraindicated groups), permethrin-based MDA, or ‘standard-care’ (where all community members were screened and referred for treatment if diagnosed with scabies). The trial found that at 12 months after MDA, the prevalence of scabies reduced in the ivermectin group by 94% (from 32% to 2%), in the permethrin group by 62% (42% to 16%) and in the standard-care group by 49% (37% to 19%). Once again, a considerable reduction was observed in the prevalence of impetigo without additional use of antibiotic therapy, most notably in the ivermectin group, where impetigo prevalence fell by 67% (25% to 8%).

Similar results were seen in two recent studies in the Solomon Islands. A before-and-after study evaluated a single round of ivermectin-based MDA, offered to a population of 26,000, in combination with azithromycin MDA for trachoma control. The co-administration was well tolerated with no serious adverse events [36]. A smaller study, from a different region of the Solomon Islands, reported a reduction in scabies prevalence of greater than 90%, 12 months after ivermectin-based MDA [37].

However, in a single cohort study in a remote island community of Australia, ivermectin-based MDA was less effective. Two MDA campaigns were implemented, 12 months apart, for a population of approximately 1000. Scabies prevalence fell from a baseline of 4% to less than 1%, 6 months after MDA, then increased to 9% after 12 months. A second MDA was conducted at this time, and scabies prevalence fell to 2% when measured 6 months later. Factors identified that may have contributed to the results included a lower than anticipated baseline prevalence, a highly mobile population with many new arrivals to the community after the first MDA, and a cluster of new cases associated with a case of crusted scabies.

Ivermectin-MDA programs for other NTDs also provide opportunities to study the effect on scabies [38]. In Zanzibar, Tanzania, annual MDA with ivermectin and albendazole for lymphatic filariasis from 2001–2005 resulted in a 68–98% reduction in clinic presentations for scabies [39]. However, in a recent, prospective study from the Kongwa District of Tanzania, where the baseline prevalence of scabies was 4.4%, annual ivermectin MDA for lymphatic filariasis was associated with a smaller reduction in scabies prevalence, to 2.9% after 4 years [40]. The authors concluded that a greater effect on scabies prevalence may not have been observed because of the relatively low prevalence and because only a single dose was given.

In addition to these community control strategies, there is increasing evidence of the use of MDA for management of scabies outbreaks within closed institutions such as schools, prisons, hospitals, aged care facilities and refugee and displaced person camps [30,41,42,43]. Scabies outbreaks in these settings are often difficult to diagnose and manage [44,45,46]. Ivermectin-based MDA has also been employed in the control of large-scale, open outbreaks, such as the current, drought-associated epidemic in Ethiopia, where more than one million people have been affected, although evaluation of this public health intervention is not currently available [47].

## 5. Outstanding Issues

The combined evidence of these control strategies reveals several common themes. First, in contrast to treating individuals and contacts, MDA strategies appear highly effective in reducing the burden of both scabies and impetigo. This strategy has been particularly successful in island communities with a very high baseline prevalence. With adequate community consultation, ivermectin-based MDA has been widely accepted by communities, well tolerated by individuals, and appeared more effective than permethrin-based MDA. Second, the benefit of MDA in communities with a lower baseline prevalence is less clear, and suggests that a different strategy may be appropriate for communities with lower prevalence (for example, less than 5%). Third, the most effective strategies have been those where active surveillance and treatment of new cases and new arrivals to the community have been incorporated, but it remains unclear to what extent these additional measures are required to ensure sustainable program success. Finally, environmental control measures (such as the washing of linen and clothing, or insecticide spraying) have not formed a component of the most successful strategies, suggesting this labor-intensive and costly strategy may not be required for community control. For control of institutional outbreaks, particularly where individuals have crusted scabies, environmental measures, if feasible, may be warranted.

Despite the promise of studies on community control for scabies, there remain several important operational questions about how their findings should be implemented, and the expected impact of such interventions. As noted, MDA appears less successful in lower-prevalence settings, and it is not clear what prevalence of scabies, or other measure, should trigger consideration of MDA. Further investigation is required to determine the optimal strategy for MDA. It is unclear whether one dose of ivermectin per MDA round is sufficient, or whether a second dose is required after 7–14 days, as is clinically recommended when using ivermectin for individual case treatment, due to its inability to kill the mite eggs [48]. The number and frequency of MDA rounds, when to stop, and duration of surveillance after stopping are all unknown. Modelling research could play an important role in this area, as for other NTDs [49,50,51]. The treatment of young children and pregnant women is critical for reducing transmission because these groups harbor a disproportionate burden of disease. However, because of current limitations on ivermectin use, they can only receive topical treatments, which are less practical. There is some evidence that ivermectin may be safe in these groups [52,53], but definitive research and development of pediatric-friendly formulations are needed. Further work on the feasibility and acceptability of MDA programs by communities in various settings is also required.

Standardized methods for diagnosis and mapping are required, possibly building on the 2018 IACS criteria, which need validation and testing in a variety of settings [26]. Investment into basic scientific research is required, including the development of diagnostic tests, ideally point of care tests for low-resource settings [54,55]. In lower-prevalence settings, or where MDA may be unrequired or infeasible, different strategies need to be developed and evaluated, including mass treatment of high-risk groups, or enhanced surveillance and intensified case management. Simplified, integrated clinical care pathways [56,57], and strategies such as teledermatology [58,59,60] may have an important role in providing high quality care to remote areas. Distinct strategies are also required for closed outbreaks in both developed and resource-limited settings. Although MDA programs do not place huge selective pressures on the microorganisms they target, ongoing monitoring for potential mite resistance to ivermectin and topical agents is important [61,62,63,64].

The impacts of scabies control programs need further evaluation, as this will inform policy makers and stakeholders of the likely health and economic benefits of the program. In particular, understanding the impact of reducing scabies and impetigo on improving quality of life and reducing downstream health complications such as skin and soft tissue infections, sepsis, and rheumatic heart disease [25]. Understanding these outcomes will facilitate cost-effectiveness evaluations in a range of settings. Research into the understanding and conception of scabies and impetigo within communities, followed by community awareness and engagement strategies, will be crucial for programmatic success.

While MDA with ivermectin appears highly effective, there are disadvantages to its use, including the apparent need for two doses, as outlined above. Moxidectin is an oral compound related to ivermectin, and was recently approved by the United States Food and Drug Administration for treatment of onchocerciasis [65]. Moxidectin has a much longer half-life than ivermectin, spanning the entire life cycle of the scabies mite [66,67]. In a preclinical treatment study using a porcine model for scabies, a single dose of moxidectin was more effective than two doses of ivermectin [68]. Clinical studies in humans are now planned, with a view to developing moxidectin as a single-dose treatment for scabies [67,69]. If clinical development is successful, moxidectin would be a very attractive option for community control of scabies because it would obviate the need for a second dose and reduce the risk of early re-infestation. Alternative topical treatments may also be required if resistance to permethrin becomes problematic [64,70].

Finally, scabies control should not occur in a vacuum. There are a number of opportunities for integration of scabies control with other NTDs, especially around the use of ivermectin [71,72]. Examples include successful co-mapping of scabies prevalence with other NTDs [17,73], and co-administration of ivermectin and azithromycin MDA with evaluation of multiple endpoints [36,37]. The roll-out of triple therapy (ivermectin, diethylcarbamazine, and albendazole) for the control of lymphatic filariasis [74,75] presents further opportunities to integrate evaluation of impact of this strategy on scabies.

## 6. Conclusions

In 2018, WHO recognized the need for ‘a global strategy for scabies control’ [24]. Such a strategy would align with the United Nations Sustainable Development Goals, including Target 3.3 to end the epidemic of NTDs (among other diseases) by 2030 [76]. Community control will be a central component of such a strategy, but broader progress will require consideration of several interrelated issues, including: engagement with key stakeholders at national, regional, and global levels to develop a coordinated, international framework; integration with existing NTD programs to leverage efficiency and cost-effectiveness of scabies control; provision of guidelines for monitoring and evaluation of programs; securement of drug supply at large scale; development of funding partnerships; and advancement of a research agenda, including operational research as outlined above, and research into improved diagnostics, new treatments (including moxidectin), and mite resistance monitoring.

## Figures and Tables

**Table 1:** Summary of 2018 IACS ^1^ criteria for the diagnosis of scabies [26].

Level	Criteria
A. Confirmed scabies	At least one of the following: A1: Mites, eggs or faeces on light microscopy of skin samples A2: Mites, eggs or faeces visualized on individual using high-powered imaging device A3: Mite visualized on individual using dermoscopy
B. Clinical scabies	At least one of the following: B1: Scabies burrows B2: Typical lesions affecting male genitalia B3 Typical lesions in a typical distribution and two history features
C. Suspected scabies	One of the following: C1: Typical lesions in a typical distribution and one history feature C2: Atypical lesions or atypical distribution and two history features
History features	H1: Itching H2: Close contact with an individual who has itching or typical lesions in a typical distribution
*Notes:*	*Diagnosis can be made at one of the three levels (A, B, or C)* *A diagnosis of clinical or suspected scabies should only be made if differential diagnoses are considered less likely than scabies.*

^1^ IACS: International Alliance for the Control of Scabies.
